# Physical Activity and Mental Well-Being Among University Students: The Role of Beliefs in the Mental Health Benefits of Physical Activity

**DOI:** 10.3390/healthcare14070955

**Published:** 2026-04-06

**Authors:** Dragan Glavaš, Marija Šakić Velić, Patrik Grubor

**Affiliations:** University Department of Psychology, Catholic University of Croatia, 10000 Zagreb, Croatia; marija.sakic@unicath.hr (M.Š.V.); pgrubor@unicath.hr (P.G.)

**Keywords:** physical activity, mental well-being, beliefs, students

## Abstract

**Background/Objectives:** In an effort to clarify the mechanisms underlying the relationship between physical activity (PA) and mental well-being, emerging evidence points to a potentially important role of beliefs about PA’s impact on mental health. Nevertheless, research in this area remains scarce. This cross-sectional study examined whether belief in the mental health benefits of PA mediates the relationship between PA level and mental well-being among university students. **Methods:** A total of 339 university students, aged 18–28, completed the Godin–Shephard Leisure-Time Physical Activity Questionnaire, the Warwick–Edinburgh Mental Wellbeing Scale, and a newly developed Belief in the Mental Health Benefits of Physical Activity Scale. **Results:** Structural equation modelling indicated a non-significant direct effect of PA level on mental well-being. However, a significant indirect effect was observed, with higher PA level being associated with stronger belief in the mental health benefits of one’s own PA, which in turn was related to better mental well-being. **Conclusions:** The findings suggest that PA level is indirectly associated with mental well-being through belief in the mental health benefits of PA among university students. These findings highlight the potential importance of PA-related beliefs in mental health promotion and point to indirect psychological pathways that may link PA and mental well-being, warranting further longitudinal examination.

## 1. Introduction

The beneficial effects of physical activity (PA) on mental health are well established [1,2], but the processes and mechanisms through which PA may exert these benefits remain poorly understood. The present study focuses on expectations and beliefs about the effects of PA as potential pathways through which it may influence mental health. Early insights into the role of expectations and beliefs in the relationship of PA with physical and mental health originate from two landmark experimental studies. Namely, Crum and Langer [3] demonstrated that merely informing people that their daily work qualified as sufficient exercise produced beneficial physical effects, despite no change in their actual activity levels. Desharnais et al. [4] found that participants in an exercise program, who were informed that the program was intended to improve psychological well-being, showed a significant increase in self-esteem, whereas this was not the case in the control group without such manipulated expectations. These findings suggested that beliefs shaped by placebo mechanisms can enhance the psychological effects of exercise, and Lindheimer et al. [5] estimated that placebo beliefs account for approximately 50% of the psychological benefits derived from exercise.

However, subsequent experimental studies failed to confirm such strong effects of placebo beliefs. Stanforth et al. [6] reported no notable placebo effect of informing participants that their work is exercise on health outcomes other than blood pressure. Arbinaga et al. [7], in a study incorporating a no-exercise control group, as well as exercise groups with and without manipulated expectations about the psychological benefits of exercise, found that exercise improved self-esteem and well-being across all groups, but heightened expectations did not yield additional psychological benefits.

The inconsistencies across study findings regarding the effects of placebo beliefs about PA on psychological outcomes can be explained by methodological and conceptual considerations. Arbinaga et al. [7] underscored the methodological challenges in disentangling the effects of beliefs from those of PA itself, especially in experimental designs that manipulate both PA and expectancies. The conceptual considerations pertain to the formation and nature of beliefs, as well as their effects on physical and psychological reactions and outcomes. Theoretical frameworks that offer plausible explanations regarding these mechanisms are Kirsch’s response expectancy theory [8,9] and Bandura’s social cognitive theory [10]. Based on the general examination of placebo effects, Kirsch [8,9] suggested that our expected involuntary responses directly produce both objective physiological changes and related subjective experiences. Bandura [10] posited that outcome expectations, defined as beliefs regarding the anticipated personal consequences of one’s actions, influence outcomes or psychological changes. These two theoretical frameworks also provide useful insights into the formation of beliefs, especially for experimental studies that aim to manipulate them. Specifically, Bandura [10] contended that merely notifying participants of potential benefits does not ensure their acceptance of or belief in those benefits, particularly when such information conflicts with their personal experiences. Kirsch [8,9] similarly emphasises that expectancy, shaped by prior experience rather than external suggestions alone, serves as a central mechanism of placebo effects.

In this context, Glavaš and Pavela Banai [11] attempted a different approach to measuring and conceptualizing beliefs. In their study, beliefs were not manipulated but instead assessed using a self-report measure of the perceived beneficial impact of one’s own PA on mental health. Their results showed that, beyond PA’s direct effect on reduced depression and anxiety symptoms among young adults, belief in PA’s mental health benefit played a key mediating role, such that higher PA levels contributed to stronger beliefs in its benefits, and these stronger beliefs were in turn associated with lower aforementioned symptoms. Although this study contributed to understanding the role of beliefs about the mental health benefits of PA by examining their natural variation in real-life contexts, it relied on a single-item measure of such beliefs. Hence, these results require further verification using a more comprehensive measure of beliefs about the benefits of PA for mental health.

The present study contributes to the research on the relationship between PA and mental well-being in at least two important ways. Firstly, a preliminary psychometric evaluation of a newly developed self-report measure assessing beliefs about the mental health benefits of personal PA is conducted. Secondly, this more comprehensive measure is used in the examination of the mediating role of beliefs about the mental health benefits of personal PA in the relationship between PA and mental well-being. Additionally, these relations are examined among university students, who are, due to various changes and challenges they are facing during this life period, a population at greater risk for poorer mental well-being and more susceptible to mental health problems [12,13,14]. Thus, examining the mechanisms by which PA may protect and enhance their mental well-being is also of practical importance.

The following hypotheses are proposed: (H1) higher PA level will be associated with stronger beliefs in the mental health benefits of one’s own PA, and (H2) beliefs about the mental health benefits of one’s own PA will mediate the relationship between PA level and mental well-being among university students.

## 2. Materials and Methods

### 2.1. Participants

The study sample consisted of N = 339 university students (80.2% female), aged 18–28 years (M = 22.23, SD = 2.02), recruited via an invitation letter outlining the research project, which specified the inclusion of healthy students capable of performing typical physical activities. In total, 195 students (57.5%) were enrolled in undergraduate programmes or the first three years of integrated study programmes, whereas 144 (42.5%) were enrolled in graduate programmes or in the later years of integrated study programmes.

### 2.2. Study Design

The study used a cross-sectional design.

### 2.3. Instruments

#### 2.3.1. Physical Activity (Godin–Shephard PA Score)

Physical activity level was assessed with the Godin–Shephard Leisure-Time Physical Activity Questionnaire (GSLTPAQ) [15], which was back-translated to Croatian by Glavaš and Pavela Banai [11]. Participants reported the frequency of their engagement in strenuous (e.g., running, jogging, football), moderate (e.g., fast walking, tennis), and mild (e.g., yoga, bowling) physical activities by responding to the question “During a typical 7-day period (week), how many times, on average, do you do the following kinds of exercise for more than 15 min?”. Following the standard scoring procedure, a composite leisure-time PA score (Leisure Score Index, LSI) was calculated by multiplying the reported frequencies of strenuous, moderate, and mild activities by their corresponding weights (9, 5, and 3), and summing the weighted scores. The LSI reflects self-reported leisure-time physical activity (PA) and represents the overall PA level. Higher scores indicate greater engagement in PA. Evidence for the scale’s validity in distinguishing between less and more physically active individuals is provided by studies showing that GSLTPAQ scores are correlated with indicators of physical fitness [16].

#### 2.3.2. Mental Wellbeing

The Short Warwick–Edinburgh Mental Wellbeing Scale (SWEMWBS) [17] was used to assess mental well-being. The 7-item version, a shortened form of the original 14-item scale, was back-translated to Croatian. Participants reported their thoughts and feelings over the past two weeks (e.g., “I’ve been dealing with problems well”) on a scale from 1 (“none of the time”) to 5 (“all of the time”). The total raw score was obtained by summing the item scores, with higher scores indicating better mental well-being. Although the developers of SWEMWBS provide a Rasch-based conversion of raw scores to metric scores [17], raw sum scores were used, as they were considered appropriate for the present analytical purpose. Given that the primary analyses were conducted using latent variable modelling, the use of raw scores was not expected to meaningfully affect the estimation or interpretation of associations among constructs. The scale’s internal consistency was high (Cronbach’s α = 0.85). Despite its brevity, the short version of this scale has demonstrated acceptable validity and reliability in studies involving samples of young people aged 15–21 [18,19] and the general population [20].

#### 2.3.3. Belief in the Mental Health Benefits of Physical Activity

Beliefs about the mental health benefits of personal PA were assessed using a newly developed measure, namely the Belief in the Mental Health Benefits of Physical Activity Scale. The development procedure and the final version of the scale used in the present study are described below.

##### Scale Development Process

The scale was developed to assess individuals’ beliefs about the mental health benefits of their own PA. Given that the scale was developed and psychometrically evaluated within a single sample, the findings reported below should be considered preliminary and require independent cross-validation. An initial pool of 16 items was generated based on empirical findings demonstrating associations between PA and various indicators of mental health, including reduced stress, improved mood and affect, enhanced coping with everyday challenges, greater self-efficacy and resilience, and better overall mental health outcomes [21,22,23,24,25]. Item generation was also conceptually informed by theoretical perspectives on the expected psychological consequences of behaviour. Specifically, the item content reflects the concept of outcome expectations within social cognitive theory [10], response expectancies regarding anticipated psychological effects of behaviour [8,9], and theoretical accounts emphasising affective responses to PA as an important component of PA experiences [26]. These perspectives informed the identification of relevant experiential domains of perceived psychological benefits, rather than serving as a direct source for item derivation.

Guided by empirical findings and informed by the theoretical perspectives outlined above, the items were designed to capture several related experiential domains of perceived psychological benefit associated with personal PA. These included beliefs about stress reduction, improvements in affective states, enhanced coping with everyday challenges, and broader contributions of personal PA to psychological functioning. Accordingly, the items addressed perceived benefits of PA, including reduction in stress and worry, positive affect, coping-related efficacy and confidence, mindful or balanced psychological functioning, and broader mental health.

Following an initial conceptual review by the research team, items exhibiting substantial conceptual overlap or redundancy were removed, resulting in an 11-item version retained for further evaluation. To assess the content validity of the 11-item scale, six experts in psychology from two universities independently assessed the relevance of each item to the construct of belief in the mental health benefits of personal PA. Experts rated item relevance using a four-point scale (1 = “not relevant”, 2 = “slightly relevant”, 3 = “sufficiently relevant”, 4 = “very relevant”). Consistent with standard content validity procedures [27,28,29], ratings of 3 (“sufficiently relevant”) and 4 (“very relevant”) were treated as indicating item relevance. Item-level content validity indices (I-CVI) were calculated as the proportion of experts rating an item as either “sufficiently relevant” or “very relevant” divided by the total number of experts. The results indicated high perceived relevance of the items: five items had an I-CVI of 0.83, while six had an I-CVI of 1.00. The average scale-level content validity index (S-CVI), calculated as the average of the I-CVI values across items, was 0.92 for the 11-item version, indicating strong overall content validity. Detailed item-level results are presented in Supplementary File S1.

A pilot study was subsequently conducted to evaluate item clarity. The pilot sample consisted of 70 adult participants (61% female; mean age = 26.76 years), including students (40%), employed individuals (56%), and a small proportion of unemployed participants (4%). Participants were asked to assess how clearly they understood each statement using a five-point clarity scale ranging from 1 (“very unclear”) to 5 (“very clear”). The results indicated a high level of item clarity. Across items, between 82.9% and 98.6% of participants rated the statements as either relatively clear or very clear, with mean clarity ratings ranging from 4.33 to 4.81 (see Supplementary File S1).

Following the content validation and pilot testing phases, the psychometric structure of the 11-item scale was further examined to evaluate its dimensionality and potential item redundancy, and to determine whether a more parsimonious item set could adequately represent the construct. As reported in the Psychometric Properties section of the Results, this process resulted in a final six-item version of the scale demonstrating satisfactory psychometric properties. Notably, these retained items also showed high levels of clarity in the pilot evaluation, with the percentage of participants rating them as relatively clear or very clear ranging from 90.0% to 97.1%, and mean clarity ratings ranging from 4.46 to 4.73 on the five-point scale (see Supplementary File S1). The retained items also demonstrated strong content validity, with the scale-level content validity index (S-CVI) increasing from 0.92 for the initial 11-item version to 0.97 for the final six-item version, indicating that content validity was maintained following the reduction in items. The psychometric structure of the scale was subsequently examined in the main study sample, and the results of these analyses are reported in the Psychometric Properties section of the Results. The scale development procedures were conducted as a part of the present study to evaluate the scale’s psychometric properties within the same sample. Accordingly, the psychometric findings should be considered preliminary and require independent cross-validation in future research.

##### Final Scale Used in the Present Study

The final version of the Belief in the Mental Health Benefits of Physical Activity Scale consists of six items reflecting perceived psychological benefits of one’s own PA. The retained items represent several experiential domains, including affective improvement (e.g., reduced worry, improved mood), cognitive functioning (e.g., maintaining concentration), mindfulness-related awareness (being present in the moment), motivational benefits, and self-confidence. Participants rated their agreement with the stem statement “I believe that the level/amount of my physical activity…” followed by six items describing potential psychological benefits. Responses were recorded on a 7-point Likert scale ranging from 1 (“strongly disagree”) to 7 (“strongly agree”). Higher scores indicate stronger beliefs that one’s own PA has beneficial effects on mental health.

The six-item version of the scale was used in all subsequent analyses. Its psychometric properties in the present sample are reported in the Psychometric Properties section of the Results.

### 2.4. Procedure

The data used in this study were collected as part of a broader research project entitled “Determinants and Mechanisms of Physical Activity’s Contribution to Mental Health”, funded by the Catholic University of Croatia. The research was approved by the Ethics Committee of the Catholic University of Croatia (Document Class: 641-03/24-03/13; No: 251-498-03-02-24-1).

The project survey was conducted online via the SurveyRock web platform (https://www.surveyrock.com; accessed on 21 July 2025) and distributed through project collaborators and partner universities across four universities, primarily via institutional mailing lists, and student networks associated with the project. Students were invited to participate via an official invitation letter that described the study’s purpose and specified that participation was intended for students who did not experience difficulties performing typical physical activities. This approach reflects a voluntary, convenience-based sampling procedure. A total of 522 students accessed the survey link, of whom 339 completed the questionnaire and were included in the final sample, yielding a completion rate of 64.9%. Only fully completed questionnaires were retained for analysis. The survey platform prevented multiple submissions from the same IP address, while IP addresses themselves were not stored in the dataset to preserve participant anonymity.

Participation in the study was voluntary, and participants were encouraged to complete the questionnaire by being informed that each completed questionnaire would result in a €1 donation from the project’s funds to a local children’s organisation. As such, the sample may be subject to self-selection bias, as participation depended on individuals’ willingness to respond to the survey invitation.

Prior to participating in the survey, participants provided informed consent on a form that described the study’s objectives, the confidentiality and anonymity of the data, and their right to withdraw from the study at any time.

### 2.5. Statistical Analysis

Statistical analyses were conducted in R (version 4.5.3.) [30]. The scale’s dimensionality was first explored using exploratory factor analysis (EFA; principal axis factoring, PAF) implemented in the psych package (version 2.6.3.) [31], which was also used to generate EFA loading diagrams. The number of factors to retain was evaluated using the Kaiser–Guttman criterion and parallel analysis. The hypothesised unidimensional structure was subsequently evaluated with confirmatory factor analysis (CFA) using lavaan (version 0.6.21) [32]. CFA figures were produced with semPlot (version 1.1.8.) [33] and semptools (version 0.3.3) [34]. Model fit was assessed using multiple complementary indices (CFI, TLI, RMSEA, and SRMR). Because the chi-square test is well known to be sensitive to sample size, model adequacy was judged primarily on the overall configuration of these indices relative to widely used cutoff guidelines [35], rather than on chi-square significance alone.

To further evaluate the psychometric properties of the belief in the mental health benefits of PA scale, additional indices relevant to internal consistency, redundancy, and convergent validity were examined, including Cronbach’s alpha, corrected item–total correlations, average inter-item correlation (AIC), composite reliability (CR), average variance extracted (AVE), and a SEM-based omega estimate derived from the CFA model.

Next, descriptive statistics (means, standard deviations, and ranges) were calculated for the observed variables, followed by Pearson’s correlation coefficients to examine associations among PA level, beliefs about the mental health benefits of PA, and mental well-being.

To test the direct and indirect associations between PA level and mental well-being via beliefs about the mental health benefits of PA, a structural equation mediation model was estimated in which PA level was specified as an observed predictor, beliefs about the mental health benefits of PA as a latent mediator, and mental well-being as a latent outcome. PA level was treated as an observed variable because the GSLTPAQ score represents a weighted composite of mild, moderate, and strenuous activity frequencies, reflecting a formative scoring logic rather than a reflective latent measurement model. Model parameters were estimated using maximum likelihood (ML), and missing-data handling was specified using full information maximum likelihood (FIML). However, all analyses were conducted on complete cases only (N = 339), and no missing responses were present in the analysed dataset. Standard errors and bias-corrected and accelerated (BCa) 95% bootstrap confidence intervals were obtained from 2000 resamples. No covariates were included in the mediation model, as the analysis was designed to examine the primary associations among the study variables.

Because all the constructs were assessed using self-report measures at a single time point, the potential influence of common method variance was additionally examined using Harman’s single-factor test. Specifically, unrotated principal component analysis (PCA) and principal axis factoring (PAF) were conducted on all the items included in the substantive model (PA level items, belief in the mental health benefits of PA items, and mental well-being items) to examine the proportion of variance accounted for by the first general factor. This procedure was conducted for both the initial 11-item belief scale and the shortened 6-item version.

## 3. Results

### 3.1. Descriptive Statistics and Correlation Analysis

Descriptive statistics and correlations among the study variables, namely physical activity (PA) level, belief in mental health benefits of PA, and mental well-being, are presented in Table 1.

Correlation analyses (Table 1) revealed a significant positive relationship among PA level, belief in the mental health benefits of PA, and mental well-being. A higher PA level was associated with stronger belief in the mental health benefits of PA (r = 0.27, *p* < 0.01), indicating that more active students had greater confidence in these benefits. The results also showed a modest correlation between PA level and mental well-being (r = 0.12, *p* < 0.05), suggesting that students engaging in more vigorous PA reported somewhat better mental well-being. Stronger belief in the mental health benefits of PA was associated with better mental well-being (r = 0.27, *p* < 0.01), suggesting that this belief is related to higher mental well-being scores. Since all variables were assessed using self-report measures, the observed correlations, particularly between conceptually related constructs, may be partly influenced by shared method variance. This is particularly relevant for the association between belief in the mental health benefits of PA and mental well-being, as both constructs reflect internal psychological states reported within the same measurement context.

The psychometric properties of the Belief in the Mental Health Benefits of Physical Activity Scale are examined in the following section.

### 3.2. Psychometric Properties of the Belief in the Mental Health Benefits of Physical Activity Scale

Psychometric properties of the Belief in the Mental Health Benefits of Physical Activity Scale were evaluated using exploratory and confirmatory factor analyses, complemented by additional diagnostics addressing potential item redundancy and shared method variance. Because the scale was developed and evaluated within a single sample, all reported psychometric findings and conclusions should be considered preliminary, should be interpreted with caution, and require independent cross-validation before firm conclusions about the scale’s structure and validity can be drawn.

#### 3.2.1. Psychometric Evaluation of the 11-Item Version

For the original 11-item version, construct validity was examined using both exploratory and confirmatory factor analyses. An exploratory factor analysis (EFA) was conducted using principal axis factoring (PAF). Based on the Kaiser–Guttman criterion, a single factor was extracted, explaining 75.7% of the variance in the observed items (λ_1_ = 8.32). Communalities ranged from h^2^ = 0.656 to h^2^ = 0.878, with standardised loadings ranging from λ = 0.81 to λ = 0.94. The EFA solution is presented in Supplementary File S2. In addition, parallel analysis supported retaining one factor. Next, the one-factor structure was evaluated using confirmatory factor analysis (CFA) with maximum likelihood estimation. The initial one-factor model showed the following fit indices: χ^2^(44) = 206.79, *p* < 0.001; CFI = 0.962; TLI = 0.953; RMSEA = 0.104 (90% CI [0.090, 0.119]); SRMR = 0.023. Because RMSEA was the only index suggesting poorer fit, modification indices were inspected and indicated that allowing the residual covariance between Item 1 (“It reduces my stress” [Smanjuje moju razinu stresa]) and Item 2 (“It reduces my worry” [Smanjuje moju zabrinutost]) would improve model fit. This specification is theoretically warranted because both items share highly overlapping affective content (stress/worry) and are phrased in an almost parallel manner, which can introduce shared item-specific variance beyond the general latent factor (i.e., local dependence due to similar wording and closely related outcomes). The revised model is presented in Supplementary File S3. Standardised factor loadings ranged from λ = 0.79 to λ = 0.91, while the residual covariance between Items 1 and 2 was 0.42. Fit indices for the revised model were: χ^2^(43) = 146.68, *p* < 0.001; CFI = 0.976; TLI = 0.969; RMSEA = 0.084 (90% CI [0.070, 0.100]); SRMR = 0.018. Taken together, this pattern of indices supports an adequate one-factor solution for the full scale, with excellent incremental fit (CFI/TLI) and residual-based fit (SRMR), alongside a substantially improved RMSEA that approaches conventional cutoffs. Composite reliability and convergent validity were additionally supported, with a composite reliability (CR) of 0.971 and an average variance extracted (AVE) of 0.754.

#### 3.2.2. Item Redundancy Diagnostics and Scale Reduction

To further evaluate the possibility of item redundancy implied by extremely high internal consistency, we computed additional indices for the 11-item version. Internal consistency was very high (Cronbach’s α = 0.971). The average inter-item correlation was also very high (AIC = 0.755), and corrected item–total correlations were uniformly strong (r = 0.799–0.921), indicating substantial homogeneity and overlap among items. Reliability was additionally quantified using a SEM-based omega estimate derived from the CFA model; omega was similarly very high (ω = 0.968), consistent with a highly coherent (and potentially redundant) item set. Because all constructs were assessed via self-report at a single time point, we also conducted a Harman single-factor test on all items entering the substantive model (three PA level items, eleven belief in the mental health benefits of PA items, and seven mental well-being items). The first unrotated component accounted for 43.51% of the variance (PCA), and the first unrotated factor accounted for 42.04% (PAF), suggesting that a single general factor did not account for the majority of covariance across the full set of measures, which is commonly interpreted as indicating that common method variance is unlikely to substantially bias the observed associations.

Given evidence of very high item homogeneity within the 11-item belief scale, a shortened 6-item version was derived to preserve content coverage while reducing redundancy. Item reduction was guided by a combination of statistical indicators (very high internal consistency and inter-item correlations) and conceptual considerations regarding semantic overlap between the items. Given the very high item homogeneity observed in the 11-item set, items were removed when they substantially overlapped in meaning with other items or functioned as overly broad, summary statements. Specifically, b1 “Reduces my level of stress” [Smanjuje moju razinu stresa] was removed due to strong conceptual overlap and similar wording with item b2 “Reduces my worry” [Smanjuje moju zabrinutost]. Item b4 “Helps me feel calmer and relaxed” [Pomaže mi da se osjećam smirenije i opuštenije] was removed because it largely reiterates the affective relief domain already covered by retained affect items. Item b6 “Helps me cope with problems and challenges” [Olakšava mi nositi se s problemima i izazovima] was excluded as a broad, umbrella-type statement that may overlap with multiple more specific outcomes. Item b8 “Helps me clear my mind” [Pomaže mi razbistriti um] was removed due to semantic overlap with retained cognitive/mindfulness-related items. Finally, item b11 “Overall, it has a positive effect on my mental health” [Općenito, pozitivno djeluje na moje mentalno zdravlje] was excluded because it represents a global appraisal that closely mirrors what the total score is intended to capture. The retained 6-item short form includes items b2 “Reduces my worry”, b3 “Increases my motivation to carry out everyday tasks” [Povećava moju motivaciju za obavljanjem svakodnevnih zadataka], b5 “Helps me maintain concentration” [Pomaže mi u održavanju koncentracije], b7 “Improves my mood” [Poboljšava moje raspoloženje], b9 “Helps me be present in the moment” [Olakšava mi biti prisutan u trenutku], and b10 “Increases my self-confidence and belief in myself” [Povećava moje samopouzdanje i vjeru u sebe], thereby maintaining a balanced representation of affective, cognitive-functional, mindfulness-related, and self-confidence benefits, while reducing content duplication.

#### 3.2.3. Psychometric Evaluation of the 6-Item Version

Psychometric evaluation of the 6-item version also supported a unidimensional structure. The shortened scale was re-examined within the same sample to confirm that the reduced item set retained this unidimensional structure. EFA using PAF retained one factor explaining 72.3% of variance (λ_1_ = 4.340), with standardised loadings ranging from λ = 0.793 to λ = 0.878 and communalities ranging from h^2^ = 0.630 to h^2^ = 0.772. Parallel analysis likewise supported retention of one factor. The EFA solution for the shortened scale is presented in Supplementary File S2. CFA indicated good model fit: χ^2^(9) = 31.65, *p* < 0.001; CFI = 0.987; TLI = 0.978; RMSEA = 0.086 (90% CI [0.055, 0.120]); SRMR = 0.015, with standardised loadings ranging from λ = 0.792 to λ = 0.880, as presented in Supplementary File S3. Internal consistency remained high (Cronbach’s α = 0.939). Although item homogeneity remained substantial, it was somewhat lower than on the full scale (AIC = 0.722), and corrected item–total correlations remained strong (r = 0.768–0.845). A SEM-based omega estimate derived from the CFA model was ω = 0.939, indicating high reliability for the short form. Composite reliability and convergent validity were also supported (CR = 0.940; AVE = 0.723). Finally, repeating the Harman single-factor test using the shortened belief in the mental health benefits of PA item set together with the other constructs in the substantive model yielded lower proportions explained by the first factor (PCA: 34.40%; PAF: 31.23%), consistent with reduced shared variance attributable to item overlap. Overall, the 6-item short form retained strong evidence of unidimensionality and high reliability while reducing redundancy relative to the original 11-item version, as reflected in slightly lower inter-item correlations and reduced shared variance in the Harman single-factor test.

### 3.3. SEM Mediation Analysis

To examine the mediating role of belief in the mental health benefits of PA in the relationship between PA level and mental well-being, we tested a structural equation model in which PA level was specified as an observed predictor, belief in the mental health benefits of PA as a latent mediator, and mental well-being as a latent outcome. PA level was treated as an observed variable because the GSLTPAQ index represents a weighted composite of mild, moderate, and strenuous activity frequencies, reflecting a formative scoring logic rather than a reflective latent measurement model. Accordingly, the three activity categories are not expected to function as interchangeable indicators of a single latent factor or to exhibit uniformly high intercorrelations. Model parameters were estimated using maximum likelihood (ML) with full information maximum likelihood (FIML) estimation for missing data, while standard errors and bias-corrected and accelerated (BCa) 95% bootstrap confidence intervals were obtained from 2000 resamples.

**Figure 1:**
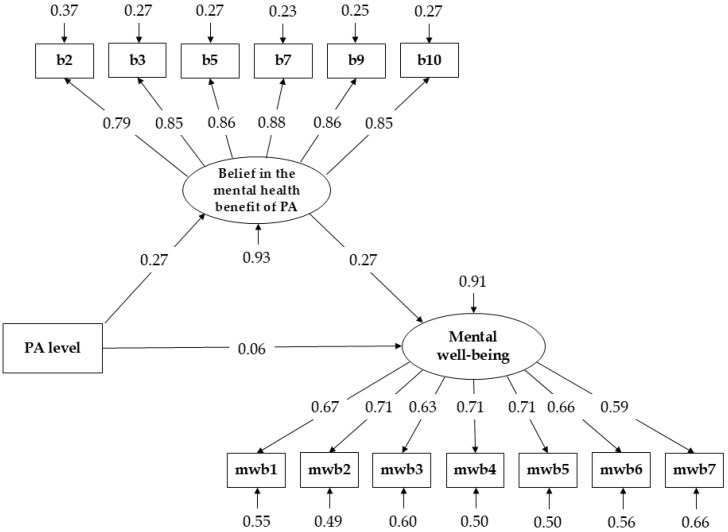
The mediating role of belief in the mental health benefits of PA in the relationship between PA level and mental well-being. Note. PA—Physical activity. Standardised path coefficients are shown; 95% confidence intervals are reported in the text.

The model demonstrated acceptable fit to the data, χ^2^(75) = 185.27, *p* < 0.001, CFI = 0.957, TLI = 0.948, RMSEA = 0.066, 90% CI [0.054, 0.078], SRMR = 0.052. All indicators loaded significantly on their respective latent factors (belief indicators: standardised λ = 0.795–0.878; well-being indicators: standardised λ = 0.586–0.711). A graphical representation of the SEM mediation model with standardised coefficients is shown in Figure 1.

The estimated structural paths indicated that a higher PA level was associated with stronger belief in the mental health benefits of PA (a = 0.013, SE = 0.003, *p* < 0.001, β = 0.271, BCa 95% CI [0.007, 0.019]). Stronger beliefs were, in turn, positively associated with mental well-being (b = 0.129, SE = 0.040, *p* = 0.001, β = 0.271, BCa 95% CI [0.055, 0.215]). The direct effect of PA level on mental well-being was not significant (c′ = 0.001, SE = 0.002, *p* = 0.392, β = 0.057, BCa 95% CI [−0.002, 0.004]). However, the indirect effect of PA level on mental well-being through beliefs in the mental health benefits of PA was significant (ab = 0.002, SE = 0.001, *p* = 0.009, BCa 95% CI [0.001, 0.003], standardised indirect effect β = 0.073). The standardised indirect effect was small in magnitude (β = 0.073). The total effect was also significant (c = 0.003, SE = 0.001, *p* = 0.034, standardised total effect β = 0.130, BCa 95% CI [0.000, 0.006]), indicating that the association between PA level and mental well-being was primarily conveyed through individuals’ beliefs about the mental health benefits of their own PA. The model explained 7.3% of the variance in beliefs (R^2^ = 0.073) and 8.5% of the variance in mental well-being (R^2^ = 0.085).

## 4. Discussion

As hypothesised, the study findings suggest that the belief in the mental health benefits of one’s own PA mediates the relationship between PA level and mental well-being among university students. While the direct effect of PA on mental well-being was not significant, a significant indirect effect was observed, with higher PA being associated with a stronger belief in the PA’s mental health benefits, which in turn correlated with better mental well-being. This mediation pattern partially aligns with the findings of Glavaš and Pavela Banai [11], which indicated that, alongside a direct association, belief in the benefits of PA mediated the association between greater PA level and lower anxiety and depression symptoms. However, our results support an indirect-only mediation model, suggesting that belief in the mental health benefits of PA may represent one pathway through which PA level is related to mental well-being. The observed correlation between belief in the mental health benefits of PA and mental well-being was modest (r = 0.27), suggesting that the constructs are related, but empirically distinct. Furthermore, the present findings may contribute to advancing our understanding of the role of beliefs in real-world contexts, addressing limitations of experimental expectancy manipulations in isolating these effects. While experimental studies relying on expectancy manipulations have yielded mixed results [3,7], the self-report approach used in this study captures naturally occurring beliefs shaped by personal PA experiences, thereby helping bridge the gap between experimental control and real-world belief formation. Thus, despite the limitations of correlational designs, this methodology may help address certain limitations of experimental designs, in which uncertainty persists about whether beliefs elicited by informing participants about PA’s benefits were genuinely internalised.

The current findings align with recent work on the activity adequacy mindset [36], which reflects beliefs about whether one’s PA suffices for health benefits. This mindset has been shown to predict significant health outcomes. Zahrt and Crum [37] found that, after adjusting for objectively measured PA, perceived PA levels predicted mortality risk, with individuals viewing themselves as less active than peers facing substantially higher risk. Similarly, in a longitudinal randomised trial using fitness trackers, Zahrt et al. [38] have shown that accurate step count feedback fostered healthier diets, improved mental health, and greater aerobic capacity (although reduced functional health was also observed), whereas deflated counts led to poorer dietary choices, reduced self-esteem, and elevated blood pressure and heart rate. Zahrt and Crum [37] also demonstrated that recommendations endorsing lower PA levels elicited a more adaptive activity adequacy mindset, enhanced self-efficacy, and engagement compared with higher-target recommendations.

In line with these findings, our indirect-only mediation model suggests that self-reported beliefs about the mental health benefits of personal PA may function as a domain-specific extension of the activity adequacy mindset among students. However, unlike the activity adequacy mindset, which concerns whether one’s PA level is adequate for overall health, our construct focuses specifically on the distinct perceived mental health benefits of personal PA. From this perspective, these beliefs may represent a more specific mental-health-oriented appraisal of the adequacy of personal PA, which may help clarify how PA relates to students’ mental well-being.

### 4.1. Theoretical Implications

The findings of this study are in line with the assumptions of Bandura’s social cognitive theory [10] and Kirsch’s response expectancy theory [8,9], and underscore the utility of these frameworks in clarifying health-directed behaviours, the underlying beliefs, and the resulting outcomes. In the context of later social cognitive accounts of health behaviour [39], the absence of a direct association between PA and mental well-being is consistent with the view that the psychological benefits of PA emerge, at least in part, through individuals’ beliefs about its mental health consequences, rather than solely through behaviour per se. The mediation pattern observed in our results supports the mechanisms and processes suggested within these theoretical frameworks, although with certain distinctions between the concept of beliefs in the mental health benefits of one’s own PA, outcome expectations, and response expectancies. Specifically, our construct captures domain-specific beliefs about mental health benefits derived from personal PA experiences. These beliefs align with, but are distinct from, outcome expectations [10,40], which involve broader cost–benefit evaluations that primarily motivate future behaviours. They also differ from response expectancies [8,9], which directly generate automatic subjective responses. Thus, beliefs in the mental health benefits of one’s own PA can be seen as specific positive outcome expectations regarding the effects of PA on psychological outcomes, most likely shaped by personal experiences. Once formed, they may operate through mechanisms similar to response expectancies, contributing to the mental well-being benefits foreseen in such anticipations.

Furthermore, these mechanisms can be framed within Williams’ integrated model of physical activity, affect, and adherence [26], which outlines the relationships between PA level, affective responses, anticipated affect, and long-term engagement. The model posits that interoceptive cues, as bodily sensations arising from higher PA intensities, especially when self-paced rather than externally imposed, shape affective responses both during and after PA. PA performed at individually optimal intensities is likely to foster more favourable affective experiences. Affective reactions subsequently inform anticipated affect, shaping expectations about thoughts and feelings during and after future PA, a process that Williams [26] suggests is the central mechanism driving long-term adherence. From this perspective, the mediation pattern observed in our study suggests an additional cognitive–affective pathway in which increased PA is associated with more positive global appraisals of its mental health benefits, which, in turn, relate to better mental well-being.

### 4.2. Study Limitations and Future Directions

While this study provides additional empirical insight into the mechanism linking PA with mental well-being, several limitations should be noted. Firstly, the study is based on a cross-sectional design, which does not enable causal inferences, nor adequate testing of potential circular or bidirectional associations among PA level, belief in its beneficial effects, and mental well-being. Thus, reverse effects are also possible, with better mental well-being and stronger belief in PA’s mental health benefits fostering more intense engagement in PA. Secondly, the measures of all the variables are based on self-reports. The advantages of assessing beliefs about the mental health benefits of PA using self-reports have been elaborated, but the overall study findings must be interpreted with consideration of potential shared method variance and consistency bias effects. To examine this issue, Harman’s single-factor test was conducted, which indicated that no single factor accounted for the majority of the variance in the data. Nevertheless, because conceptually related constructs were assessed concurrently, using the same method, some degree of shared method variance or partial construct overlap cannot be fully ruled out. Consequently, the observed associations between variables may be partly inflated due to the shared measurement method. As the key mediation pathway involves conceptually related self-report constructs (beliefs and mental well-being), this association may be particularly susceptible to inflation due to shared method variance. However, the modest magnitude of this association (r = 0.27) suggests that the constructs are related but not redundant. Moreover, the indirect-only mediation pattern, including the absence of a direct association between PA level and mental well-being, is less consistent with a generalised inflation effect that would be expected to affect all paths in a similar manner. Furthermore, Harman’s single-factor test is known to have limited sensitivity for detecting common method variance and therefore provides, at best, a coarse diagnostic. In this context, the observed pattern of associations provides a complementary basis for evaluating the potential impact of method-related bias. Taken together, these considerations suggest that, although common method variance cannot be fully ruled out, it is unlikely to fully account for the observed pattern of relationships. Some inflation of specific associations, particularly between beliefs and mental well-being, may nevertheless remain possible. Future research would benefit from combining self-report measures with objective PA indicators and multi-method assessments to further reduce potential method-related bias. Thirdly, although an indirect-only mediation pattern was observed in the results, suggesting that belief in the mental health benefits of one’s own PA may represent an important mechanism through which PA is associated with mental well-being, it should be noted that the amount of variance in mental well-being explained by this belief is relatively modest. Specifically, the model explained only 7.3% of the variance in belief about the mental health benefits of PA and 8.5% of the variance in mental well-being. Thus, although statistically significant, the contribution of PA level and these beliefs to explaining differences in students’ mental well-being appears limited. Finally, the study sample is predominantly female, which limits the generalizability of the findings. This distribution likely reflects the composition of respondents who chose to participate, as well as the disciplinary context of the participating institutions, which primarily included social study programs in social sciences, where female students are more strongly represented. Accordingly, the findings may be more representative of female respondents, and their applicability to male student populations should be interpreted with caution. In addition, only basic participant characteristics were recorded, and potentially relevant contextual variables (e.g., socioeconomic status or academic stress) were not assessed, although such factors may also be associated with students’ PA and mental well-being.

Future studies should utilise longitudinal designs to clarify the direction and dynamics of the relationships among PA, belief in its beneficial effects on mental health, and mental well-being. Williams’s [26] model offers a solid theoretical and heuristic framework, particularly for longitudinal studies employing models such as the random-intercepts cross-lagged panel models. These models could help explain circularity and the relative strength of specific directional relationships. Social cognitive theory [10,39,40] also holds promise for integrating theoretically relevant constructs, including self-efficacy, into these models. Research by Egele et al. [41] with young to middle-aged adults, and by White et al. [42] with middle-aged and older adults, demonstrates the predictive validity of self-efficacy for future PA behaviour. Thus, further studies should examine its role in these potentially circular processes linking intensity and types of PA, beliefs about its effects, and mental well-being, as its role within such dynamic systems remains poorly understood and is scarcely explicitly modelled. A comprehensive integration of self-efficacy into longitudinal frameworks may clarify its role in amplifying, mediating, or stabilising these reciprocal relationships over time. Additionally, future studies could benefit from including objective measures of PA and a wider range of mental health indicators, as well as other psychological and contextual factors that may affect students’ mental well-being, and examining these relations in more diverse samples.

This study also provides initial evidence for the validity of the Belief in the Mental Health Benefits of Physical Activity Scale. Future research should implement comprehensive psychometric procedures, including forward–backward translation approaches, to ensure linguistic and conceptual equivalence across diverse populations. Because both exploratory and confirmatory factor analyses were conducted within the same sample, the reported factor structure should be considered preliminary and requires cross-validation in independent samples. Further validation steps should also include multi-sample invariance testing across diverse populations, examination of discriminant validity against conceptually related constructs, and evaluation of predictive validity against objective health markers, thereby enhancing the applicability of this measure.

### 4.3. Practical Implications

The findings of this study have several practical implications in the area of health promotion, particularly among university students. However, these implications should be considered in light of the study’s methodological constraints related to the design, sample, and model specification. PA has consistently been identified as an effective means of improving students’ mental well-being [43]. Our finding that belief in the mental health benefits of PA may represent one of the mechanisms through which PA contributes to mental well-being indicates that strengthening these beliefs may help enhance the mental health benefits of PA. Therefore, preventive and intervention activities aimed at increasing PA among university students could benefit from incorporating modifications of their cognitive appraisals and expectations about PA’s mental health outcomes. For example, belief-oriented interventions, such as providing evidence-based information on the psychological effects of PA, can be integrated into existing curricula and PA promotion programs for students.

Secondly, the initial development of the Belief in the Mental Health Benefits of Physical Activity Scale is an important step toward a more reliable assessment of these beliefs. While the current evidence is based on preliminary validation and does not permit definitive conclusions about its psychometric properties, the scale provides a valuable foundation for future refinement and application, using more diverse samples.

Finally, these findings highlight the need for a comprehensive approach to mental health promotion that integrates behavioural, cognitive, and affective elements. Public health policies aimed at enhancing PA levels should consider complementing behavioural recommendations with initiatives to modify beliefs and expectations related to PA. Such an approach could amplify the effectiveness of PA campaigns by engaging both behavioural and psychological pathways to improved mental well-being.

## 5. Conclusions

This study contributes to our understanding of the mechanisms and processes linking PA and mental well-being. The findings suggest that the link between mental well-being and PA is indirect, with individuals’ beliefs about the mental health benefits of their PA being a significant mediator. By elucidating this psychological pathway, the study offers a more nuanced understanding of the complex relationships between PA and mental well-being, emphasising indirect associations that warrant further examination into potential causal interconnections and additional influencing factors.

## Figures and Tables

**Table 1:** Descriptive statistics and intercorrelations between PA level, beliefs in the mental health benefits of PA, and mental well-being.

	M (SD)	Range	1	2	3
PA level (1)	68.47 (26.68)	17–170	-	0.27 **	0.12 *
Belief in the mental health benefits of PA (2)	33.12 (8.07)	6–42		-	0.27 **
Mental well-being (3)	26.00 (4.39)	10–35			-

* *p* < 0.05; ** *p* < 0.01. Note. PA = physical activity; M = mean; SD = standard deviation; numbers (1–3) correspond to the variables listed in the rows.

## Data Availability

The data supporting reported results can be found as publicly archived datasets generated during the study at: https://osf.io/ybdca/files/osfstorage (accessed on 5 April 2025).

## Supplementary Materials

The following supporting information can be downloaded at: https://www.mdpi.com/article/10.3390/healthcare14070955/s1, Supplementary File S1: Item-level content validity and pilot clarity result; Supplementary File S2: Exploratory factor analyses; Supplementary File S3: Confirmatory factor analyses.
