# Supplementary material for: Physical Activity and Mental Well-Being Among University Students: The Role of Beliefs in the Mental Health Benefits of Physical Activity
Source: Healthcare (Basel). 2026 Apr 6;14(7):955. doi: 10.3390/healthcare14070955 (PMC13073164; doi:10.3390/healthcare14070955)
Supplement: Supplementary file 1 [file healthcare-14-00955-s001.zip › Supplementary File S1_Item-level content validity and pilot clarity results.docx]

**Supplementary File 1:** Item-level content validity and pilot clarity result

Belief in the Mental Health Benefits of Physical Activity Scale – Item-level content validity and pilot clarity results

Expert content validity ratings (N = 6) and pilot clarity evaluation (N = 70) of the initial 11 items of the Belief in the Mental Health Benefits of Physical Activity Scale [Skala uvjerenja o učinku tjelesne aktivnosti na mentalno zdravlje].

Items of the Belief in the Mental Health Benefits of Physical Activity Scale were preceded by the stem statement:

“I believe that the level/amount of my physical activity…”

[Uvjerenja sam da razina/količina moje tjelesne aktivnosti…]

Participants rated their agreement with each statement using a seven-point Likert scale ranging from 1 (“strongly disagree”) to 7 (“strongly agree”).

| Item, English translation [Croatian Original] | | I-CVI | % relatively/ very clear | *M* (*SD*) |  |
| --- | --- | --- | --- | --- | --- |
| 1 | Reduces my level of stress [Smanjuje moju razinu stresa] | 0.83 | 94.3 | 4.67 (0.58) |  |
| 2 | **Reduces my worry [Smanjuje moju zabrinutost]** | 1.00 | 94.3 | 4.46 (0.61) |  |
| 3 | **Increases my motivation to carry out everyday tasks [Povećava moju motivaciju za obavljanjem svakodnevnih zadataka]** | 1.00 | 92.9 | 4.64 (0.62) |  |
| 4 | Helps me feel calmer and relaxed [Pomaže mi da se osjećam smirenije i opuštenije] | 0.83 | 92.9 | 4.56 (0.63) |  |
| 5 | **Helps me maintain concentration [Pomaže mi u održavanju koncentracije]** | 1.00 | 90.0 | 4.50 (0.68) |  |
| 6 | Helps me cope with problems and challenges [Olakšava mi nositi se s problemima i izazovima] | 1.00 | 82.9 | 4.33 (0.83) |  |
| 7 | **Improves my mood [Poboljšava moje raspoloženje]** | 1.00 | 97.1 | 4.73 (0.51) |  |
| 8 | Helps me clear my mind [Pomaže mi razbistriti um] | 0.83 | 90.0 | 4.61 (0.71) |  |
| 9 | **Helps me be present in the moment [Olakšava mi biti prisutan u trenutku]** | 0.83 | 92.9 | 4.49 (0.63) |  |
| 10 | **Increases my self-confidence and belief in myself [Povećava moje samopouzdanje i vjeru u sebe]** | 1.00 | 94.3 | 4.64 (0.59) |  |
| 11 | Overall, it has a positive effect on my mental health [Općenito, pozitivno djeluje na moje mentalno zdravlje] | 0.83 | 98.6 | 4.81 (0.43) |  |
| Table S1. Belief in the Mental Health Benefits of Physical Activity Scale: Item wording, content validity, and pilot clarity results  *Note.*  Items retained in the final six-item version of the scale are shown in bold.  I-CVI = item-level content validity index. Content validity ratings were provided by six experts (N = 6), who evaluated item relevance using a four-point scale (1 = “not relevant”, 2 = “slightly relevant”, 3 = “sufficiently relevant”, 4 = “very relevant”). I-CVI values represent the proportion of experts rating an item as either “sufficiently relevant” or “very relevant”.  The scale-level content validity index (S-CVI) was 0.92 for the initial 11-item version and 0.97 for the final six-item version.  % relatively/very clear represents the percentage of participants in the pilot study (*N* = 70) selecting response options 4 (“relatively clear”) or 5 (“very clear”) on a five-point clarity scale ranging from 1 (“very unclear”) to 5 (“very clear”). *M* (*SD*) represents the mean clarity rating and standard deviation.  The scale was translated by the authors, and no back-translation procedure was performed. | | | | |  |
|  |  |  |  |  |  |
|  |  |  |  |  |  |
|  |  |  |  |  |  |
