# Supplementary material for: Physical Activity and Mental Well-Being Among University Students: The Role of Beliefs in the Mental Health Benefits of Physical Activity
Source: Healthcare (Basel). 2026 Apr 6;14(7):955. doi: 10.3390/healthcare14070955 (PMC13073164; doi:10.3390/healthcare14070955)
Supplement: Supplementary file 1 [file healthcare-14-00955-s001.zip › Supplementary file S2_Exploratory factor analyses.docx]

**Supplementary File S2**: Exploratory factor analyses


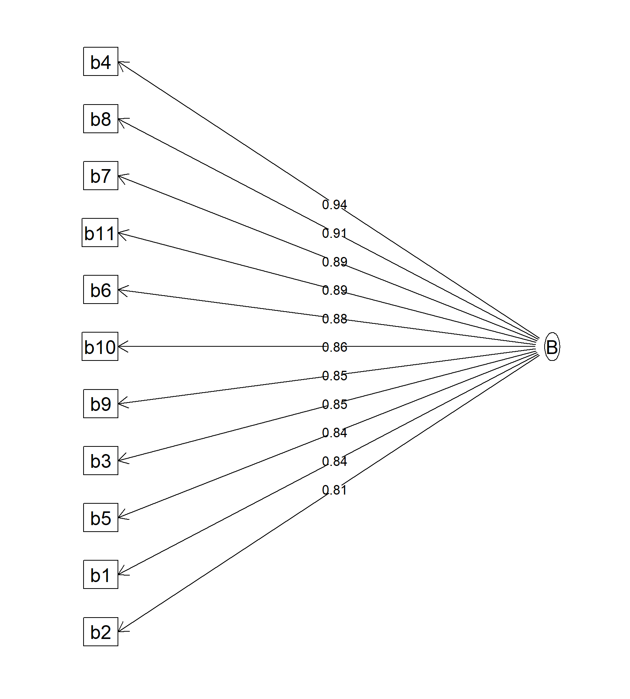


Figure S1. Exploratory factor analysis of the 11-item Belief in the Mental Health Benefits of Physical Activity Scale (*N* = 339)

*Note*. B = belief latent factor; b1–b11 = scale items; λ = standardised factor loadings (0.81–0.94). The first factor explained 75.7% variance (λ₁ = 8.33). Item communalities ranged from h² = .656–.878.


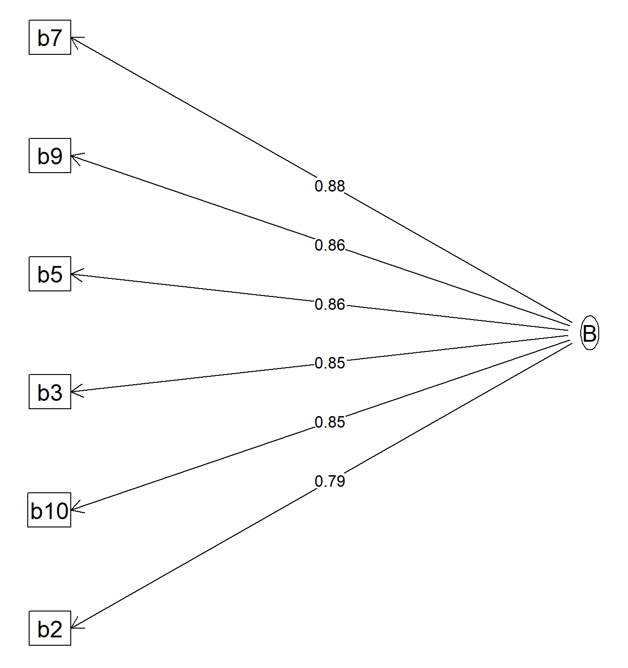


Figure S2. Exploratory factor analysis of the six-item Belief in the Mental Health Benefits of Physical Activity Scale (N = 339).

*Note*. B = belief latent factor; retained items: b2, b3, b5, b7, b9, and b10. λ = standardized factor loadings (.793–.878). The first factor explained 72.3% of the variance (λ₁ = 4.340). Item communalities ranged from h² = .630 to .772
