# Supplementary material for: Physical Activity and Mental Well-Being Among University Students: The Role of Beliefs in the Mental Health Benefits of Physical Activity
Source: Healthcare (Basel). 2026 Apr 6;14(7):955. doi: 10.3390/healthcare14070955 (PMC13073164; doi:10.3390/healthcare14070955)
Supplement: Supplementary file 1 [file healthcare-14-00955-s001.zip › Supplementary file S3_Confirmatory factor analyses.docx]

**Supplementary File S3:** Confirmatory factor analyses


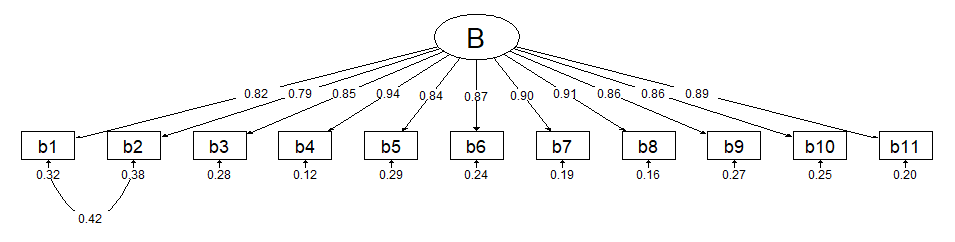


Figure S3. Confirmatory factor analysis model with residual covariance for the 11-item Belief in the Mental Health Benefits of Physical Activity Scale.

*Note*. B = belief latent factor; b1–b11 = scale items; λ = standardized factor loading; e = measurement error. Standardized loadings ranged from λ = .79 to .91. A residual covariance between b1 (“reduces my stress”) and b2 (“reduces my worry”) was estimated (r = .42) to account for shared item-specific variance due to highly overlapping affective content and nearly identical wording. Model fit: χ²(43) = 146.68, p < .001; CFI = .976; TLI = .969; RMSEA = .084; SRMR = .018.


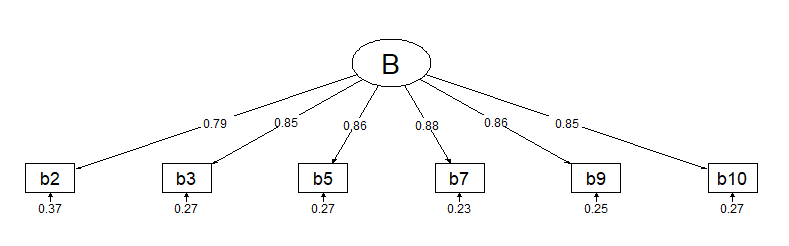


Figure S4. Confirmatory factor analysis model of the six-item Belief in the Mental Health Benefits of Physical Activity Scale.

*Note*. = B = belief latent factor; retained items: b2, b3, b5, b7, b9, and b10; λ = standardized factor loading; e = measurement error. Standardized loadings ranged from λ = .792 to .880. Model fit: χ²(9) = 31.65, p < .001; CFI = .987; TLI = .978; RMSEA = .086; SRMR = .015.
